# Radiomics Analysis on Contrast-Enhanced Spectral Mammography Images for Breast Cancer Diagnosis: A Pilot Study

**DOI:** 10.3390/e21111110

**Published:** 2019-11-13

**Authors:** Liliana Losurdo, Annarita Fanizzi, Teresa Maria A. Basile, Roberto Bellotti, Ubaldo Bottigli, Rosalba Dentamaro, Vittorio Didonna, Vito Lorusso, Raffaella Massafra, Pasquale Tamborra, Alberto Tagliafico, Sabina Tangaro, Daniele La Forgia

**Affiliations:** 1 Istituto Tumori “Giovanni Paolo II” I.R.C.C.S., 70124 Bari, Italy; lilianalosurdo@gmail.com (L.L.); v.didonna@oncologico.bari.it (V.D.); v.lorusso@oncologico.bari.it (V.L.); massafraraffaella@gmail.com (R.M.); pasqualetamborra@gmail.com (P.T.); d.laforgia@oncologico.bari.it (D.L.F.); 2 Dip. Interateneo di Fisica “M. Merlin”, Università degli Studi di Bari “A. Moro”, 70125 Bari, Italy; teresamaria.basile@uniba.it (T.M.A.B.); roberto.bellotti@uniba.it (R.B.); 3 Dip. di Scienze Fisiche, della Terra e dell’Ambiente, Università degli Studi di Siena, 53100 Siena, Italy; ubaldo.bottigli@unisi.it; 4 Dip. di Scienza della Salute, Università degli Studi di Genova, 16132 Genova, Italy; alberto.tagliafico@unige.it; 5 INFN—Istituto Nazionale di Fisica Nucleare, 70126 Bari, Italy; Sonia.Tangaro@ba.infn.it

**Keywords:** breast cancer, radiomics analysis, feature extraction, feature selection, Haar wavelet decomposition, gray-level co-occurrence matrix, contrast-enhanced spectral mammography

## Abstract

Contrast-enhanced spectral mammography is one of the latest diagnostic tool for breast care; therefore, the literature is poor in radiomics image analysis useful to drive the development of automatic diagnostic support systems. In this work, we propose a preliminary exploratory analysis to evaluate the impact of different sets of textural features in the discrimination of benign and malignant breast lesions. The analysis is performed on 55 ROIs extracted from 51 patients referred to Istituto Tumori “Giovanni Paolo II” of Bari (Italy) from the breast cancer screening phase between March 2017 and June 2018. We extracted feature sets by calculating statistical measures on original ROIs, gradiented images, Haar decompositions of the same original ROIs, and on gray-level co-occurrence matrices of the each sub-ROI obtained by Haar transform. First, we evaluated the overall impact of each feature set on the diagnosis through a principal component analysis by training a support vector machine classifier. Then, in order to identify a sub-set for each set of features with higher diagnostic power, we developed a feature importance analysis by means of wrapper and embedded methods. Finally, we trained an SVM classifier on each sub-set of previously selected features to compare their classification performances with respect to those of the overall set. We found a sub-set of significant features extracted from the original ROIs with a diagnostic accuracy greater than 80%. The features extracted from each sub-ROI decomposed by two levels of Haar transform were predictive only when they were all used without any selection, reaching the best mean accuracy of about 80%. Moreover, most of the significant features calculated by HAAR decompositions and their GLCMs were extracted from recombined CESM images. Our pilot study suggested that textural features could provide complementary information about the characterization of breast lesions. In particular, we found a sub-set of significant features extracted from the original ROIs, gradiented ROI images, and GLCMs calculated from each sub-ROI previously decomposed by the Haar transform.

## 1. Introduction

In image processing, feature extraction plays a very important role: it allows obtaining quantitative information (features) from medical images that cannot be detected by means of simple visual observation by the operator, using appropriate mathematical methods and computers [1]. This discipline, known as radiomics, is an emerging translational research topic in cancer studies. Radiomics analysis on different medical images is often inserted in a framework of pattern recognition tasks for characterizing lesions of various natures and on different imaging modalities [2,3]. Indeed, although clinicians are trained for visual pattern recognition, it is still a subjective evaluation. Several studies have investigated the usefulness and reliability of radiomics to discriminate benign breast lesions from cancers, evaluate prognosis, or response to therapies, demonstrating that it could potentially improve diagnosis and characterization of lesions: automatic recognition tools can provide objective information to support clinical decision-making or improve the radiologist’s confidence in the challenging diagnostic task [4,5].

For this purpose, many techniques may be applied. Depending on the clinical utility of the research and the type of medical images, different typologies of features can be extracted from them, such as statistical, textural, morphological, and shape features [6,7,8,9], each of which provides particular information useful to describe a specific aspect of a lesion. Some radiomics works are based on texture analysis, since the texture may be defined as the pattern of information or arrangement of structure found in an image [10,11,12].

Texture analysis aims to describe the fundamental characteristics of textures and to represent them in a simpler, but distinctive form, in order to use them for a robust and accurate classification and segmentation of objects [13]. There are two types of textural feature measurements, first and second order: in the first order, texture measurements are statistics directly calculated from an individual pixel and do not consider pixel neighbor relationships (i.e., the intensity histogram and intensity features); in the second order, measurements consider the relationship between neighboring pixels. Moreover, when the image is analyzed and decomposed into different frequency sub-bands by means of wavelet transforms or co-occurrence matrices, this technique may be more effective [13,14].

In general, this approach can catch information about the characteristics of a tumor missed by a human reader, therefore providing details with a significant diagnostic value. Textural features capture spatial and spectral frequency patterns, as well as characterize the relationships between different intensity levels within the lesion; they might not be immediately visible to radiologists and thus have the potential to complete their diagnostic skills. Moreover, this analysis can be performed in an automated way without any human intervention, not as well as for morphological/shape features or BIRADS [15] descriptors.

With particular reference to breast lesions, in order to characterize masses and microcalcifications, textural features are often extracted from mammographic images after having applied the so-called Gray-Level Co-occurrence Matrix (GLCM), which correlates the intensity of the gray levels of neighboring pixel pairs in different directions [16,17,18,19]. Some works also consider the possibility to decompose each image into sub-images by means of 2D and discrete wavelet transforms [18,20,21], Gabor filters [19,22,23], and the image gradient [24] before calculating co-occurrence matrices to detect defects in the image texture. Then, they make a comparison between these feature extraction methods in order to estimate the most appropriate method of feature extraction from mammograms. To analyze breast Magnetic Resonance (MR) images, textural features extracted by GLCMs [25,26] or wavelet transforms [27] are used, while in some other works, a combination of textural and statistical [28,29] or morphological [30] features is preferred.

Nevertheless, in recent years, new radiological imaging equipment has been developed in order to increase the diagnostic performances, especially when breast is dense. Among these new techniques, Contrast-Enhanced Spectral Mammography (CESM) [31,32,33] combines the principles of mammography with the injection of an intravenous iodinated Contrast Medium (CM), which allows, as in MR images, a contrastographic evaluation of the breast: this highlights the areas that capture the contrast medium, the typical expression of neo-angiogenesis neoplasm. As in MR, CESM images may present enhancement of normal breast parenchyma after CM injection, known as Background Parenchymal Enhancement (BPE) [15,34]. On the contrary, CESM is less influenced by hormonal status than MR [35], and this could provide important additional information on the detection of lesions in patients with a high BPE in degree where distinguishing a lesion from the non-enhanced background is objectively difficult. Moreover, CESM is less expensive and more tolerated by patients than MR [36].

In the literature, several analyses are aimed at comparing CESM performances to mammography [37,38,39] and MR [40,41,42] ones by the reading medical images by expert radiologists. Differently, there are few works in which a radiomics analysis has been performed. The first approaches to develop computerized algorithms addressed to increase the diagnostic performances on CESM images were reported only in [43,44,45]. In these works, several features used, such as morphological and BIRADS descriptors, presented limits of subjectivity in the feature extraction process due to the fact that they depended on the judgment of the radiologist who manually segmented the lesions and determined their benign/malignant nature based on his/her experience. Moreover, some standard textural features were extracted on original and pre-processed images by using GLCMs of original images, Gabor filter banks, and Laplacian of Gaussian histograms. However, no comparative analysis was performed between the different features used.

In our work, we propose a preliminary radiomics analysis aimed to explore the usefulness of quantitative information extracted from CESM images, both in original and pre-processed format, to support the radiologist in the diagnosis of breast cancer. Specifically, in order to make the lesion classification more objective and operator independent, once the radiologist has identified the Regions Of Interest (ROIs), the characteristic features are extracted in an automated manner. The aim of our work is to understand the behavior of each different set of well known textural features automatically extracted from CESM images and to compare them with each other. An important role is played by the feature selection processes used to describe and characterize ROIs: starting from the initial feature set, a sub-set of these features, characterized by a higher discriminating power, is selected for a more manageable data processing [46]. Then, we select the most important features by developing two different approaches of feature importance, such as embedded and wrapper methods.

## 2. Materials and Methods

### 2.1. Materials

#### 2.1.1. CESM Examination

**Figure 1:**
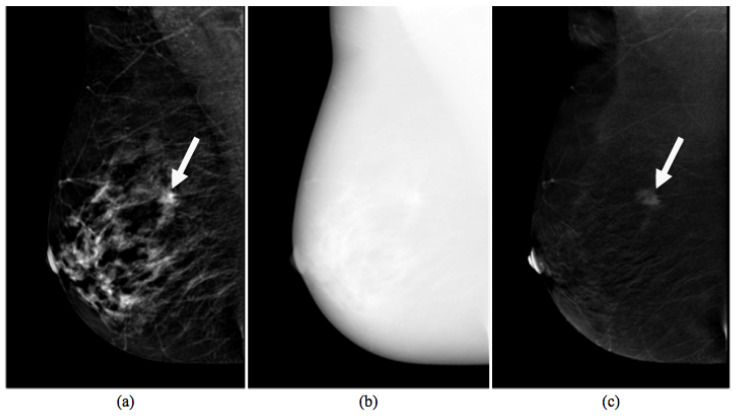
Images produced by CESM instrumentation. Typical example of low energy (**a**), high energy (**b**), and recombined (**c**) images [37]. The white arrow points to a suspicious lesion.

CESM is a technique allowing the acquisition of multiple views of both breasts by producing two types of images. A typical example of CESM images is shown in Figure 1, where it is clear that a Low Energy (LE) image may be overlapped completely by a 2D digital mammography image (a), while High Energy (HE) images are not displayable in the reporting monitor (b); instead, a ReCombined (RC) image highlights contrast medium uptake, as a breast MR image (c).

The CESM technique consists of the acquisition of low and high energy digital mammograms, both in CranioCaudal (CC) and MedioLateral Oblique (MLO) views, with the dual energy technique after the administration of an intravenous iodinated CM by an automated injector to ensure a constant flow. In order to reduce the so-called anatomical noise due to the typical tissue overlap especially of mammographically “dense” fibro-glandular breasts, a combined mammographic image, where only the CM is highlighted, is produced by means of spectral subtraction.

On these RC images, some motion blur could be sometimes observed because of movements between the acquisition of different images; however, this dual energy subtraction technique is less sensitive to movement artifacts than traditional temporal subtraction.

In this study, a modified digital mammography system derived from a standard Senographe Essential (GE Healthcare) was used for all CESM exams. CESM images were all in DICOMformat and were evaluated by two dedicated radiologists with more than 10 years of experience in reading mammography and breast MR images and trained in reading contrast enhanced images.

#### 2.1.2. Experimental Dataset

From March 2017 to June 2018, we collected CESM images of patients referred to Istituto Tumori “Giovanni Paolo II” I.R.C.C.S. of Bari (Italy) from the breast cancer screening phase. Patients undergoing CESM had indications for breast MR, but they could not perform it due to several contraindications or impossibility. Therefore, in our Institute, the use of this method is applied only as a second alternative to MR, even for patients who have to perform urgent MR for therapies or programmed surgery, but that have not found access to MR, as indicated by the European guidelines on CESM [47]. Our observational study was approved by the Medical Ethics Committee of the Institute, and written informed consent prior to undergoing CESM examination was signed by all eligible patients.

We selected images in MLO or CC view of 51 patients aged between 38 and 80 years (with a mean of 52.3±9.9 years), resulting in being positive in the methods for the presence of at least one finding after histological examination.

In order to avoid the BPE degree being a confusing factor for the purposes of evaluating the diagnostic capacity of the features, the sample was selected in such a way as to have a fair distribution of benign and malignant lesions for each BPE class.

Two of our radiologists dedicated to senologic diagnostics identified and classified a total of 55 primary and, if present, also secondary lesions (29 benign and 26 malignant) from 0.5 to 13.5 cm according to the BIRADS classification [48]: lesions belonging to BIRADS 2 and 3 classes were labeled as benign, while lesions belonging to BIRADS 4 and 5 classes were considered as malignant. Then, the histological diagnosis based on bioptic sampling established that 29 ROIs contained benign lesions and 26 ROIs included malignant ones. All ROIs were extracted both from LE and RC images.

### 2.2. Methods

#### 2.2.1. Feature Extraction

In this paper, we present a comparison of several feature sets in order to establish an order of importance among them in the classification of lesions on CESM images. For this purpose, some properties of the image texture [49], such as gray-tone distribution and spatial dependencies, were considered. MATLAB R2017a (MathWorks, Inc., Natick, MA, USA) software was used for all analysis steps.

**Figure 2:**
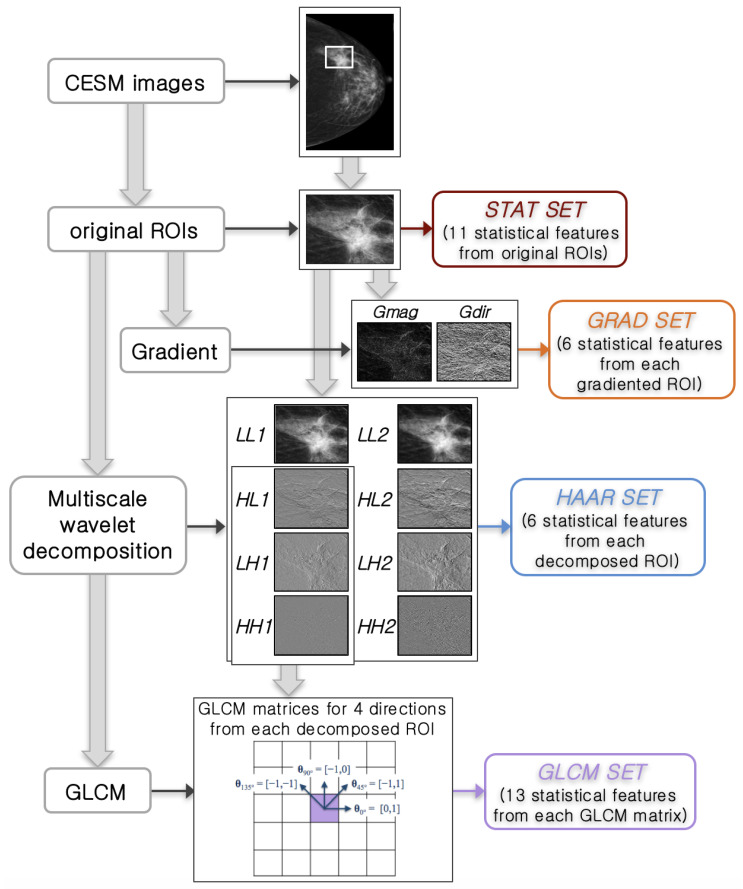
Scheme of feature extraction. Feature extraction process going from the identified original ROI to each extracted feature set. This scheme is shown starting from a low energy image, but it is also performed for recombined images.

The considered sets of features extracted from the original ROIs (both LE and RC), their gradient, and wavelet decompositions using some methods are listed below. Figure 2 summarizes the feature extraction process, from the identification of ROIs to each extracted feature set.

STAT set: Several standard statistical features (mean, standard deviation and their ratio, variance, skewness, entropy, relative smoothness, and kurtosis) were directly extracted from each original ROI. Moreover, the minimum and maximum values of gray-level and their difference were computed. These 11 features were extracted both from LE and RC original ROIs, forming a vector of 22 features and giving some relevant and objective information of each ROI.

GRAD set: The gradient of an image is represented as a two-component vector (*x*- and *y*-derivative) defined at each pixel [50]: they can be computed by the convolution with a kernel, such as the Sobel or Prewitt operator, since the image is a discrete function for which the derivatives are not defined. For each vector, the magnitude *Gmag* shows how quickly the intensity of each pixel is changing in the neighborhood of pixel (*x*, *y*) in the direction of the gradient, while the direction *Gdir* represents the orientation of greatest intensity change in the neighborhood of pixel (*x*, *y*). They are given by $\sqrt{f_{x}^{2}+f_{y}^{2}}$ and $\arctan(f_{y}/f_{x})$, respectively, where $f_{x}$ and $f_{y}$ are the components of the vector. If the original images are obtained under different conditions (i.e., exposure energy), it is possible that the pixel values are drastically different, even though they represent the same characteristics (e.g., a benign or malignant lesion). The gradiented images are less susceptible to these factors and therefore are usually used for robust feature and texture matching. For this feature set, mean, variance, skewness, entropy, relative smoothness, and kurtosis were extracted from the gradient’s magnitude and direction of each LE and RC original ROI by using a Sobel kernel, thus obtaining a total of 24 features.

HAAR set: As a fundamental property of the image texture, the scale at which the image is observed and analyzed was exploited by using a wavelet transform based on texture analysis approach, such as the Haar wavelet transform [50,51]. This allows decomposing the image by using an orthonormal basis composed by scaled and translated functions. Conceptually, the scaled function represents the low frequency component of the scaling function in 2 dimensions, obtaining one 2D scaling function. On the contrary, the translated function includes three different components (horizontal, vertical, and diagonal). Since this wavelet transform is separable, four combinations of these functions may be obtained by means of low and high filters. The Haar transform is considered the first known wavelet basis and widely used as a teaching example. In particular, the 2D Haar wavelet decomposes an image first with a low-pass filter obtaining a downscaled Low-Low (LL) sub-image and then with a high-pass filter for each component of the translated function obtaining the corresponding High-Low (HL), Low-High (LH), and High-High (HH) sub-images. In general, low and medium frequencies match image content while high ones usually emphasize noise or texture areas. Therefore, in the wavelet domain, noise and image content or image regions of different complexity (quasi-homogeneous, textural, containing borders or objects, etc.) can be distinguished and used in noise parameters’ estimation, filtering, compression, etc. Then, the image decomposition can be iterated at successive levels applying the Haar wavelet transform on the first downscaled sub-image. In this work, we performed 2D Haar transform at two levels of decomposition on each ROI; hence, we extracted mean, variance, skewness, entropy, relative smoothness, and kurtosis from each sub-ROI, both LE and RC, thus obtaining a set of 96 features.

GLCM set: The approach considers how many times the gray-level intensity value of a reference pixel is associated with another gray-level intensity value on each neighbor pixel in a specific spatial relationship obtaining the Gray-Level Co-occurrence Matrix (GLCM) [10,13] for each ROI. This spatial relationship, known as offset, is given by the distance between a pixel and its neighbors according to a specified direction ($dir1=0^{\circ }$, $dir2=45^{\circ }$, $dir3=90^{\circ }$, $dir4=135^{\circ }$).

Thus, this last set of statistical features (contrast, correlation, cluster prominence, cluster shade, dissimilarity, energy, entropy, homogeneity, sum average, sum variance, sum entropy, difference entropy, and normalized inverse difference moment) was extracted from the co-occurrence matrices of each sub-ROI previously decomposed by the Haar transform only at Level 1 (HL, LH, and HH) in the four directions, obtaining 156 features. These particular measurements have invariant properties under some image transformations because they are calculated by GLCM and allow better detecting any defects in the image texture. Then, they could determine the location and the range of the pixels having a structure with considerable deviation in their values of intensity or spatial arrangement with respect to the background texture. Since we have taken into account both LE and RC images, this set was totally formed by 312 features.

#### 2.2.2. Feature Reduction and Importance Analysis

**Figure 3:**
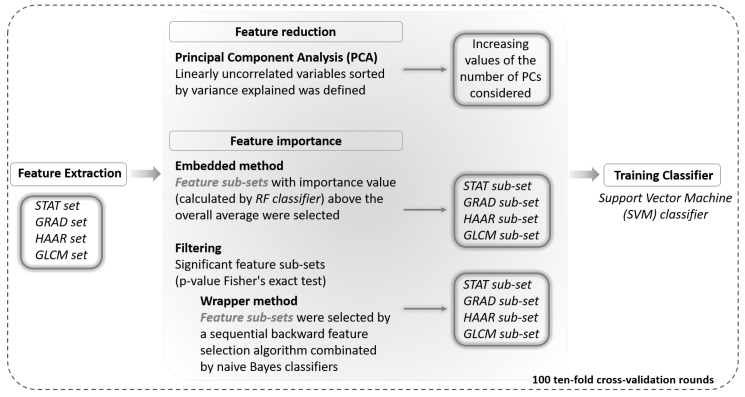
Schematic overview of the radiomic analysis approach. Textural features automatically extracted from each ROI in the first step (Figure 2) are analyzed by using a principal components analysis and a feature importance process by means of two different approaches (wrapper and embedded techniques). The discrimination performances of an SVM classifier trained on the feature subsets are evaluated on 100 ten-fold cross-validation rounds.

The aim of this work is to explore the discriminating power of feature sets extracted by different techniques, as described above, to characterize the benign and malignant breast lesions. For this purpose, we present a multi-parametric analysis approach to evaluate how these individual typologies of features behave on images of this still unexplored imaging technique (i.e., CESM). Specifically, we analyzed the features of each feature set jointly in order to solve the benign vs. malignant classification problem by means of two different approaches, reduction of the feature number and selection of the most discriminating features. In Figure 3, a schematic overview of our feature analysis approach is shown.

First, we evaluated the overall impact of each feature set on the diagnosis through a Principal Component Analysis (PCA) [52]. This method of feature reduction performs a linear mapping of the data to a lower dimensional space in such a way that the variance of the data in the low-dimensional representation is maximized, allowing removing redundant information. In this way, the number of features is reported in the same number of linearly uncorrelated latent variables: this technique performs a linear transformation of the features that projects the original ones into a new Cartesian system where the variables are sorted in descending order with respect to the overall variance percentage explained. Therefore, we evaluated the classification performance of each feature set considering an increasing number of Principal Components (PCs) to train a Support Vector Machine (SVM) classifier.

Moreover, in order to identify for each feature set extracted a sub-set of them with higher diagnostic power, we developed two approaches for the task of feature importance evaluation, such as wrapper and embedded methods [46].

Wrapper methods measure the usefulness of features based on the classifier performance. They solve the “real” problem using the predictor as a black box and its performance as an objective function to evaluate the variable sub-set [53]. The sequential backward feature selection algorithm identifies the features that have best predicted the expected result by sequentially removing features from the initial candidate set until a removal increases the error or the accuracy decreases significantly; therefore, it stops when a local maximum is found. In particular, in this work, we developed two steps of feature selection wrapper algorithm: first, we identified a significant feature sub-set correlated with the variable to be predicted (*p*-value Wilcoxon–Mann-Whitney test <0.05); then, on this selected sub-set of features, we implemented a sequential backward feature selection algorithm combined with a naive Bayes classifier.

Embedded methods allow an optimization between the interaction of the selected features and the machine learning algorithm used for the classification, because the selection criterion is grafted onto it. Indeed, they combine the qualities both of filter and wrapper methods. Random Forests (RF) are among the most popular machine learning algorithms because they generally provide good predictive performance and low over-fitting [54]. The RF classifier processes an analysis of feature importance with respect to its expected result; therefore, these methods essentially fulfill the goal, i.e., the optimization of the classification performances. In particular, the tree-based strategy used by RF naturally ranks by how well they improve the purity of the node: nodes with the greatest decrease in impurity are at the start of the trees, while nodes with the least decrease in impurity occur at the end of trees measured by Gini’s diversity index.

Thus, in this work, the RF algorithm allowed estimating predictor importance values by permuting out-of-bag observations among the trees, and features with an importance value above the overall average were selected.

For each feature set, we identified the significant feature sub-set with a selection frequency different from chance (*p*-value Fisher’s exact test <0.05). Specifically, we tested that the occurrence frequency of the features selected by each of two methods (i.e., embedded and wrapper) was significantly different from that obtained after permuting the diagnostic target in the dataset [55].

Finally, in order to compare the classification performances of the features selected by the two methods, we trained a binary SVM classifier. The performances of the prediction model were evaluated on 100 ten-fold cross-validation rounds [56] in terms of:

Accuracy=(TP+TN)/(TP+TN+FP+FN),

Sensitivity=TP/(TP+FN),

Specificity=TN/(TN+FP),

where *TP* and *TN* stand for True Positive (number of true malignant ROIs identified) and True Negative (number of true benign ROIs identified) cases, while *FP* (number of benign ROIs identified as malignant) and *FN* (number of malignant ROIs identified as benign) are the False Positive and False Negative ones, respectively.

## 3. Results

The goal of our analysis was to investigate the impact of textural features extracted in an automated manner through different techniques in discriminating benign and malignant ROIs. The main interest of this study was to understand the total behavior of these textural features in order to have an overall view of the goodness of the approach used for the feature reduction. First, we evaluated the overall discriminant power of each feature set performing a principal component analysis as the feature reduction technique. Then, for each feature set, we searched for those that contributed the most to discriminating benign and malignant ROIs.

### 3.1. Principal Component Analysis

**Figure 4:**
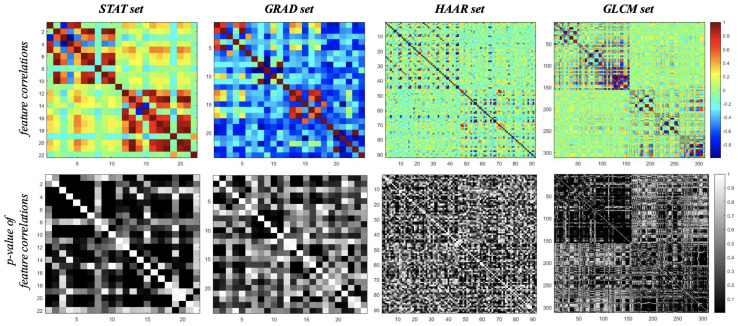
Correlation graphs. Graphs of feature correlations (**top**) and their relative *p*-values (**bottom**) for each set.

A preliminary analysis, reported in Figure 4, shows that in each feature set, there were significant, often very strong correlations between them. This suggested that a feature reduction approach can be useful to analyze the overall diagnostic power of each feature set, specifically for more numerous sets, like HAAR and GLCM sets. Therefore, we performed a PCA for each suitably standardized set of features.

**Figure 5:**
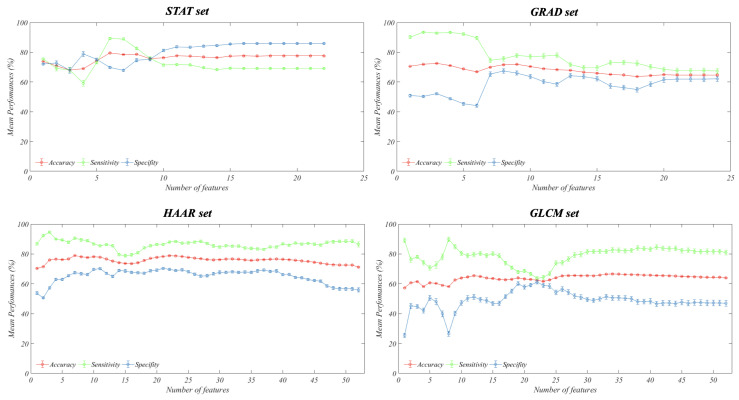
Classification performances. Mean classification performances evaluated on 100 ten-fold cross-validation rounds for each feature set with respect to the number of Principal Components (PCs) used to train an SVM classifier.

In Figure 5, we show the performance measurements obtained by training an SVM classifier for increasing values of the number of PCs used that were previously ordered with respect to the overall variance explained by the same principal components. There are several criteria in order to select this number of PCs that guarantees the lowest possible loss of information, but they could lead to different results. Since the purpose of the work was to provide an overall assessment of the diagnostic power of the extracted features and not to optimize the classification problem, we show the global performance trend.

The STAT and HAAR sets showed the best mean accuracy (about 80%) on 100 ten-fold cross-validation rounds; on the contrary, the GLCM set was the one with the lowest diagnostic power (the best mean accuracy reached about 64%), while the GRAD set achieved a maximum mean accuracy of about 72%. Specifically, in accordance with what has been observed about the presence of significant correlations between features in each set, the best predictive performance was achieved with no more than 10 principal components.

### 3.2. Feature Importance Analysis

**Figure 6:**
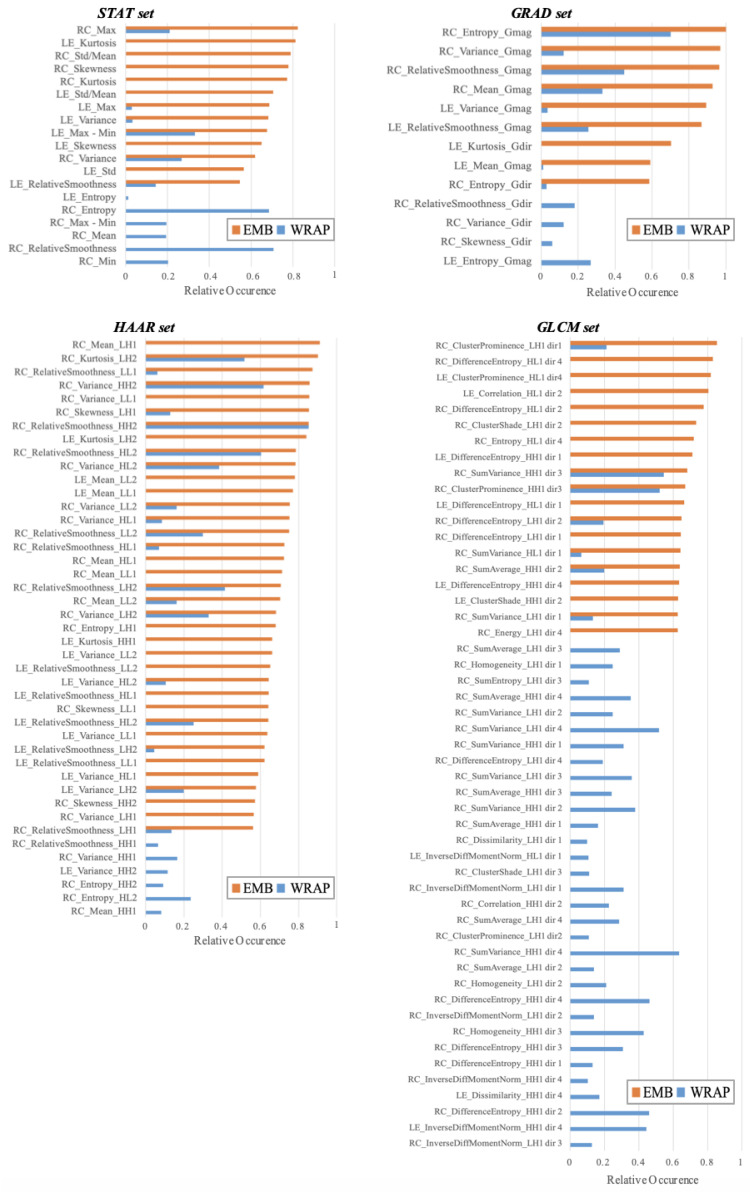
Occurrence frequency of the selected features. Occurrence frequency of the features selected by the Embedded (EMB) and Wrapper (WRAP) methods that is significantly different from chance (*p*-value of Fisher’s exact test ≤0.05) for each feature set.

In Figure 6, we summarize the significant features whose occurrence frequency was significantly different from chance by testing as described above.

Regarding the STAT set, 19 of the 22 features were statistically significant, and only six of these were selected by both methods implemented.

Among the 24 features of the GRAD set, only 13 were significant, and eight of them were selected by both implemented methods.

The HAAR and GLCM sets were the groups with the largest number of features. The HAAR set consisted of 96 features, and only 43 were statistically significant, while no more than 19 features were selected by both methods implemented. The GLCM set was that with the greater reduction in the number of selected features. Indeed, it consisted of 312 features, and only 51 resulted in being statistically significant. The features selected by the wrapper method were 39, and only seven of them were also selected by the embedded method.

The diagnostic performances of the sub-set formed by only significant features selected for each set were evaluated by training an SVM classifier and obtaining on 100 ten-fold cross-validation rounds in terms of accuracy, sensitivity, and specificity, as summarized in Table 1. We report the mean value and relative confidence interval of each performance measure obtained in the cross-validation process to provide an idea of our results’ reliability. During the study and development of the present work, we evaluated several classifiers, such as SVM, Bayes, and k-nearest neighbors, different from those used in the feature selection step, in order to have results that were not biased by the same selection technique. However, SVM was the best performing classifier; therefore, we preferred to report only this result without overloading the reading of this paper.

Generally, the significant feature sub-set selected for the STAT and GRAD sets reproduced the average performance obtained by PCA, and therefore, they were the ones with the highest discrimination power. Specifically, the feature sub-set selected with the embedded approach was preferable to that identified with the wrapper method because it reduced the trade-off between sensitivity and specificity. For the model trained on the STAT sub-set selected by the embedded method, these were 86.38% and 75.00%, respectively, with respect to the 90.28% and 71.55% obtained with features selected by the wrapper method; similarly, the model trained on the GRAD sub-set selected by the embedded method reached a sensitivity and a specificity of 81.28% and 70.76%, respectively, compared to the 82.86% and 66.45% obtained with features selected by the wrapper method. For both sets, the classification performances decreased when the model was trained on all significant features. As described above, the average classification accuracy obtained by the PCs of the GLCM set did not exceed 64%. Nevertheless, using the sub-set of significant features identified by the wrapper method, the model reached a mean accuracy of 76.50%, a mean sensitivity of 81.93%, and a mean specificity of 71.07%.

On the contrary, the HAAR set of features used globally were the ones with the highest discriminating power, but the performances reached with this set trained the model only on the significant features, collapsing by about 20 percentage points, regardless of the sub-set of features used. The best performance obtained by the HAAR set of features was reached by using the significant sub-set selected by the wrapper method with a mean accuracy, sensitivity, and specificity of 60.76%, 54.79%, and 66.72%, respectively.

## 4. Discussion

In this paper, several methods were used to extract features from CESM images and to analyze them. This automatic extraction process was performed by taking into account computational simplicity, invariance properties, and noise sensitivity. Moreover, we focused our work on textural features, since they highlighted the relationships between different levels of intensity within the lesion and captured spatial and spectral frequency patterns. For these purposes, we extracted feature sets calculating statistical measures on the original ROIs (STAT set) and on their manipulations by filters (GRAD set), wavelet functions (HAAR set), or considering the relationships between neighboring pixels and their gray levels (GLCM set).

Currently, the literature is poor regarding radiomic analysis of breast cancer on CESM images useful to develop systems as diagnostic support tools. In [43], a first approach was proposed to analyze morphological descriptors on mass (shape, margins, pattern, and degree of internal enhancement) and non-mass (distribution, pattern, and degree of internal enhancement) lesions on CESM images aimed to assess their impact on the discrimination between benign and malignant ones.

In [45], the authors developed a convolutional neural network based decision support system combining CESM pixel information with BIRADS descriptors provided by radiologists. Nevertheless, these works presented limits of subjectivity of the feature extraction process, in particular of morphological features and BIRADS descriptors, because of their dependence on the radiologist’s experience.

Instead, in [44], a multi-parametric feature analysis approach aimed to construct a computer aided diagnosis tool to increase the diagnostic performances of the CESM technique was presented. In this work, a set of morphologic and textural features was extracted by GLCMs from original images, Gabor filter banks, Laplacian of Gaussian histograms, local binary pattern, and discrete orthonormal Stockwell transform, and after that, an expert breast radiologist manually outlined lesion boundaries on each image. However, an analysis of the individual types of features used was not performed.

Our goal was to evaluate several well known textural feature sets in biomedical image analysis whose extraction does not require the intervention of radiologists, in order to find quantitative additional information that may be integrated with the experience of human readers to enhance diagnostic accuracy.

For this purpose, firstly, we evaluated the overall diagnostic power of each feature set by means of a principal component analysis. Then, we implemented two different feature selection techniques, such as wrapper and embedded; these methods are quite similar since they are used to optimize the performance of a learning algorithm or model. However, they differ in the fact that only for the embedded methods, an intrinsic model building metric is used during training, while the wrapper ones operate by iteratively selecting the insertion or removal of a feature and evaluating the results obtained. Finally, the accuracy classification of PCs calculated and also each feature subsets identified were evaluated in the cross-validation process with a well known state-of-the-art classifier, such as SVM, independent of those used in the selection phase.

The experimental results showed that for the STAT set, the wrapper method has more frequently selected features related to entropy and relative smoothness, as well as absolute variability measures; on the contrary, the embedded method has more frequently selected features linked to the shape of the gray levels’ distribution, such as kurtosis and skewness, as well as absolute and relative variability measures. With regard to the significant features of the GRAD set, it emerged that the most important features were measures calculated on the gradient magnitude of ROIs both on LE and RC images. The significant feature sub-set selected by the two approaches used in this work for STAT and GRAD sets was actually that with the highest discriminating power, because it could reproduce the average performances obtained by using it globally through the PCs.

Most of the significant features of the HAAR and GLCM sets were calculated on RC images, unlike what happened for the other two feature sets. In particular, among the 43 and 51 significant features of HAAR and GLCM, only 15 and 9 features, respectively, were calculated on LE images. For these two sets, the classification performances underwent a trend reversal when they were globally used by means of a PCA or when only the significant features identified were used. Indeed, the average classification accuracy obtained using the PCs of the GLCM set did not exceed 64%. Nevertheless, when we trained the classifier on the sub-set of significant features, the classification accuracy grew at least about 10 percentage points; therefore, there were evidently some features that introduced some distortions. Instead, the features of the HAAR set with the highest discriminating power were those used globally. However, the performances achieved in this way of training the model on the significant feature sub-set fell by about 20 percentage points. This indicated that the other features of the set contributed to a lesser extent to the resolution of the diagnostic problem.

## 5. Conclusions

Recent feasibility studies suggested that CESM is an useful investigation tool and that it can provide pre-operative staging and accurate treatment planning in breast cancer patients with an accuracy no less than MR [40]. CESM showed interesting results in terms of diagnostic sensitivity, compatible with those obtained by MR: in [39], the sensitivity was 100% for both techniques, while in [41], it was 100% by CESM and 93% by breast MR. Moreover, on the basis of state-of-the-art comparative results, CESM also had better tolerance and less discomfort than MR, as shown in [36,57,58]. Thus, this new imaging technique can represent a valid alternative to MR, also due to its better tolerance and lower discomfort with respect to the latter [36,57]. Nevertheless, CESM, as MR, has presented false positive cases [31], and it can still be considered a method that is subjective and dependent on the operator’s experience due to the current lack of objective diagnostic support systems.

In this work, we proposed some preliminary results of a radiomics analysis useful to drive future works about automatic radiological support systems for the diagnosis of breast lesions by means of CESM images. We performed an extraction process of textural features, and then, we evaluated the diagnostic power of four feature sets extracted by using different techniques.

Textural features have the potential to support the diagnostic skills of radiologists because they capture spatial and spectral frequency patterns, often not easily visible to the human reader.

We found a sub-set of significant features extracted from the original ROIs, gradiented ones, and GLCMs calculated from each sub-ROI previously decomposed by the Haar transform. Nevertheless, the feature sets extracted from each sub-ROI decomposed by two levels of Haar transform were reliable in differentiating benign from malignant breast lesions when all of these were used without any selection. Moreover, most of the significant features calculated on HAAR decompositions and their GLCMs were extracted from RC images.

Future works include a validation study in which we will test the robustness of the significant features identified in a larger population, also with respect to the histological results of each lesion. Moreover, we will develop a computer aided diagnosis system combining them in order to optimize the classification performances.

## Figures and Tables

**Table 1:** Classification performances of the SVM classifier trained on sub-sets of significant features identified by two methods of feature selection. Furthermore, the total performances obtained taking into account the significant sub-sets selected from both methods are shown.

Method of Feature Selection	Feature Set (# of Selected Features)	Accuracy (%) Mean [CI 95%]	Sensitivity (%) Mean [CI 95%]	Specificity (%) Mean [CI 95%]
Embedded	STAT (13)	80.69[80.42,80.96]	86.38[85.59,87.17]	75.00[74.10,75.90]
	GRAD (9)	76.02[75.65,76.39]	81.28[80.60,81.96]	70.76[70.19,71.33]
	HAAR (37)	59.22[58.25,60.19]	70.59[66.65,74.53]	47.86[44.18,51.70]
	GLCM (19)	73.86[73.37,74.35]	85.62[83.68,87.56]	62.10[59.96,64.24]
Wrapper	STAT (10)	80.91[80.65,81.16]	90.28[89.75,90.81]	71.55[71.09,72.01]
	GRAD (12)	74.66[73.97,75.35]	82.86[80.83,84.89]	66.45[64.44,68.46]
	HAAR (25)	60.76[59.91,61.61]	54.79[50.16,59.42]	66.72[62.24,71.20]
	GLCM (39)	76.50[75.92,77.08]	81.93[80.75,87.94]	71.07[69.67,66.22]
Embedded + Wrapper	STAT (19)	79.43[79.25,79.61]	82.83[82.10,83.56]	76.03[75.25,76.81]
	GRAD (13)	74.47[73.87,75.06]	83.17[81.49,84.85]	65.76[63.82,67.70]
	HAAR (43)	58.83[57.90,59.76]	61.76[56.94,66.58]	55.90[51.52,60.28]
	GLCM (51)	75.95[75.36,76.54]	80.59[79.20,81.98]	71.31[69.41,73.21]

## Abbreviations

The following abbreviations are used in this manuscript:

BPE	Background Parenchymal Enhancement
CC	CranioCaudal
CESM	Contrast-Enhanced Spectral Mammography
CI	Confidence Interval
CM	Contrast Medium
dir1	Direction 1 ($0^{\circ }$)
dir2	Direction 2 ($45^{\circ }$)
dir3	Direction 3 ($90^{\circ }$)
dir4	Direction 4 ($135^{\circ }$)
EMB	Embedded
FN	False Negative
FP	False Positive
Gdir	Gradient direction
Gmag	Gradient magnitude
GLCM	Gray-Level Co-occurrence Matrix
HE	High Energy
HH	High-High
HL	High-Low
LDA	Linear Discriminant Analysis
LE	Low Energy
LH	Low-High
LL	Low-Low
MLO	MedioLateral Oblique
MR	Magnetic Resonance
PC(A)	Principal Component (Analysis)
RC	ReCombined
RF	Random Forest
ROI	Region Of Interest
SD	Standard Deviation
SVM	Support Vector Machine
TN	True Negative
TP	True Positive
WRAP	Wrapper
